# Granular Carbon-Based Electrodes as Cathodes in Methane-Producing Bioelectrochemical Systems

**DOI:** 10.3389/fbioe.2018.00078

**Published:** 2018-06-12

**Authors:** Dandan Liu, Marta Roca-Puigros, Florian Geppert, Leire Caizán-Juanarena, Susakul P. Na Ayudthaya, Cees Buisman, Annemiek ter Heijne

**Affiliations:** ^1^Sub-Department of Environmental Technology, Wageningen University & Research, Wageningen, Netherlands; ^2^Fraunhofer Institute for Environmental, Safety, and Energy Technology UMSICHT, Oberhausen, Germany; ^3^Laboratory of Microbiology, Wageningen University & Research, Wageningen, Netherlands

**Keywords:** methane production, intermittent current supply, low cathode overpotential, bioelectrochemical system (BES), granular carbon-based electrode

## Abstract

Methane-producing bioelectrochemical systems generate methane by using microorganisms to reduce carbon dioxide at the cathode with external electricity supply. This technology provides an innovative approach for renewable electricity conversion and storage. Two key factors that need further attention are production of methane at high rate, and stable performance under intermittent electricity supply. To study these key factors, we have used two electrode materials: granular activated carbon (GAC) and graphite granules (GG). Under galvanostatic control, the biocathodes achieved methane production rates of around 65 L CH_4_/m^2^cat_proj_/d at 35 A/m^2^cat_proj_, which is 3.8 times higher than reported so far. We also operated all biocathodes with intermittent current supply (time-ON/time-OFF: 4–2′, 3–3′, 2–4′). Current-to-methane efficiencies of all biocathodes were stable around 60% at 10 A/m^2^cat_proj_ and slightly decreased with increasing OFF time at 35 A/m^2^cat_proj_, but original performance of all biocathodes was recovered soon after intermittent operation. Interestingly, the GAC biocathodes had a lower overpotential than the GG biocathodes, with methane generation occurring at −0.52 V vs. Ag/AgCl for GAC and at −0.92 V for GG at a current density of 10 A/m^2^cat_proj_. 16S rRNA gene analysis showed that *Methanobacterium* was the dominant methanogen and that the GAC biocathodes experienced a higher abundance of proteobacteria than the GG biocathodes. Both cathode materials show promise for the practical application of methane-producing BESs.

## Introduction

The expansion of global energy demand results in an increasing utilization of fossil fuels, which leads to unwanted CO_2_ emissions (Rogelj et al., 2016). To mitigate CO_2_ emissions, the energy transition from fossil fuels to renewable energy is necessary. In the Energy Roadmap 2050 released by European Commission in 2011, the share of renewable energy in the final gross energy consumption will grow from 10% of today, to 30% in 2030, and at least 55% in 2050 (Commission, 2011). The substantial rise of renewable electricity demand requires new technologies for electricity storage, because the renewable electricity produced is fluctuating and intermittent due to the intermittent nature of wind and sun (Hu et al., 2018).

Power to Gas (PtG) technologies have been reported as a flexible option to convert and store excess renewable electricity from the power grid (electricity) into the gas grid (CH_4_) (Bailera et al., 2017). CH_4_ can be generated by reduction of CO_2_ through thermochemical or biological methanation. Methane-producing bioelectrochemical systems (BESs) are one form of biological methanation (Geppert et al., 2016). In methane-producing BESs, H_2_O is typically used as an electron donor, and oxidized at the anode (Van Eerten-Jansen et al., 2012). At the cathode, CO_2_ is reduced to CH_4_ by microorganisms. This assembly of cathode and microorganisms is called a biocathode.

Since the concept of methane-producing BESs has been shown in 2009 (Cheng et al., 2009), methane-producing BESs have mainly been studied at constant external electricity supply. The electricity generated by the renewable sources is, however, intermittent. So far, performance of biocathodes under intermittent electricity supply has not been studied. Intermittent operation has been performed with capacitive anode electrode materials in the form of activated carbon granules (GAC) for wastewater microbial fuel cells (MFCs) (Borsje et al., 2016; Santoro et al., 2017). These capacitive bioanodes can store electrons generated by electroactive microorganisms in the charging period (open circuit), and afterwards, these stored electrons could be harvested in the discharging cell (closed circuit) (Deeke et al., 2015). Use of granular electrodes with this capacitive property (storage of electrons) might benefit methane-producing BESs operated with intermittent electricity supply, so that the capacitance can act as an electron buffer when current peaks occur.

Besides the capacitance property of GAC, use of granular carbon-based electrodes is in general beneficial to the performance of biocathodes. The reason behind this may be that carbon-based materials have good biocompatibility, and the 3D granular structure can provide benefits for the attachment of microorganisms and increase mass transfer between the bulk solution and the electrode (Guo et al., 2015; Jourdin et al., 2015). In addition, GAC has been proven to stimulate methane production in anaerobic digestion, as it probably promotes direct interspecies electron transfer from *Geobacter* (Liu et al., 2012)*, Sporanaerobacter, and Enterococcus* (Dang et al., 2016) species to methanogens. Addition of pre-acclimated GAC as inoculum has also been shown to enhance methane production and decrease startup time in the methane-producing BESs, although carbon brushes were used as cathode electrode (LaBarge et al., 2017).

In this paper, we report the use of GAC and graphite granules (GG) in a packed bed as the cathode electrode. Three intermittent current supply modes with time-ON/time-OFF (4–2′, 3–3′, and 2–4′) were performed at two different current densities (10 and 35 A/m^2^ cat_proj_). The effect of intermittent current supply with different time intervals was studied. We tested both granule types in duplicate reactors for 137 days and assessed performance in terms of methane production rate and current-to-methane efficiency. We also analyzed the microbial community composition.

## Materials and methods

### Experimental setup

We operated four bioelectrochemical reactors (see Figure S1 in the Supporting Information). Each reactor contained an anodic and cathodic chamber, each with a volume of 33 cm^3^ (11 × 2 × 1.5 cm). A cation exchange membrane (FumaTech GmbH, Ingbert, Germany) was used with a projected surface area of 22 cm^2^ (11 × 2 cm). As cathode materials, we used GAC with a specific surface area of 764 m^2^/g (Cabot Norit Nederland B.V., Zaandam, the Netherlands; 1–3 mm diameter) and GG with a specific surface area of 0.438 m^2^/g (Carbone Lorraine Benelux BV, Wemmel, Belgium; 3–5 mm), leading to a substantially higher capacitance property in GAC compared with GG (Borsje et al., 2016).

Two cathodic chambers were packed with GAC granules (GAC_1_ with 8.5 g and GAC_2_ with 8.4 g). The other two cathodic chambers were packed with GG granules (GG_1_ with 26 g and GG_2_ with 29.2 g). All the granules were washed by distilled water before use. The current collector at the cathode was a plain graphite plate. The projected cathode surface area was 22 cm^2^ (11 × 2 cm), and was equal to the membrane surface area. The granule bed was tightly packed to ensure good contact between granules and current collector. The anodic chambers contained a 22-cm^2^ platinum-iridium-coated titanium plate as electrode (Magneto Special Anodes BV, Schiedam, the Netherlands). The anodic chambers were filled with glass beads with a 7-mm diameter (Hecht-Assistent, Sondheim v. d. Rhön, Germany) to further ensure tight packing of the carbon granules. The reference electrodes (3 M KCl Ag/AgCl, QM710X, QIS, Oosterhout, the Netherlands, +0.205 V vs. standard hydrogen electrode) were connected to the anolyte and catholyte solutions. Throughout this paper, all potentials are expressed against Ag/AgCl reference electrode.

Each cathodic chamber was connected to a liquid-gas separation bottle (60 mL) with a gas bag of 2 L (Cali-5-Bond^TM^). After the separation bottle, the catholyte was channeled into the recirculation bottle (500 mL), where CO_2_ was sparged. The excess CO_2_ went through a water lock and was released into the environment. All four anode chambers shared the same anolyte that was pumped via a recirculation bottle (5 L). Anolyte and catholyte flow rates were 7 mL/min.

### Electrolytes and microorganisms

The catholyte consisted of a 50 mM phosphate buffer (2.77 g/L NaH_2_PO_4_·2H_2_O and 4.58 g/L Na_2_HPO_4_) with 0.2 g/L NH_4_Cl, 0.13 g/L KCl, 10 mL/L Wolfe's vitamin solution and 10 mL/L Wolfe's modified mineral solution (Wolin et al., 1963). Catholyte pH was 7.1. The fresh catholyte was flushed with N_2_ gas for 30 min before each use. In order to keep the catholyte with sufficient CO_2_ and stable pH simultaneously, the catholyte in the recirculation bottle was sparged with CO_2_ for 2 h/day during weekdays. After day 71, the catholyte was sparged with CO_2_ continuously.

All cathode chambers were inoculated with 10 mL of an anaerobic mixed culture (volatile suspended solids = 12.9 ± 1.3 g/L), which contained 50% anaerobic granular sludge from the paper industry wastewater treatment facility in Eerbeek (the Netherlands) and 50% anaerobic sludge from the municipal wastewater treatment facility in Ede (the Netherlands).

The anolyte consisted of a 50 mM phosphate buffer at pH 7. The anolyte was continuously flushed with N_2_ gas in the recirculation bottle to keep O_2_ levels at a minimum.

### System operation

**Figure 1:**
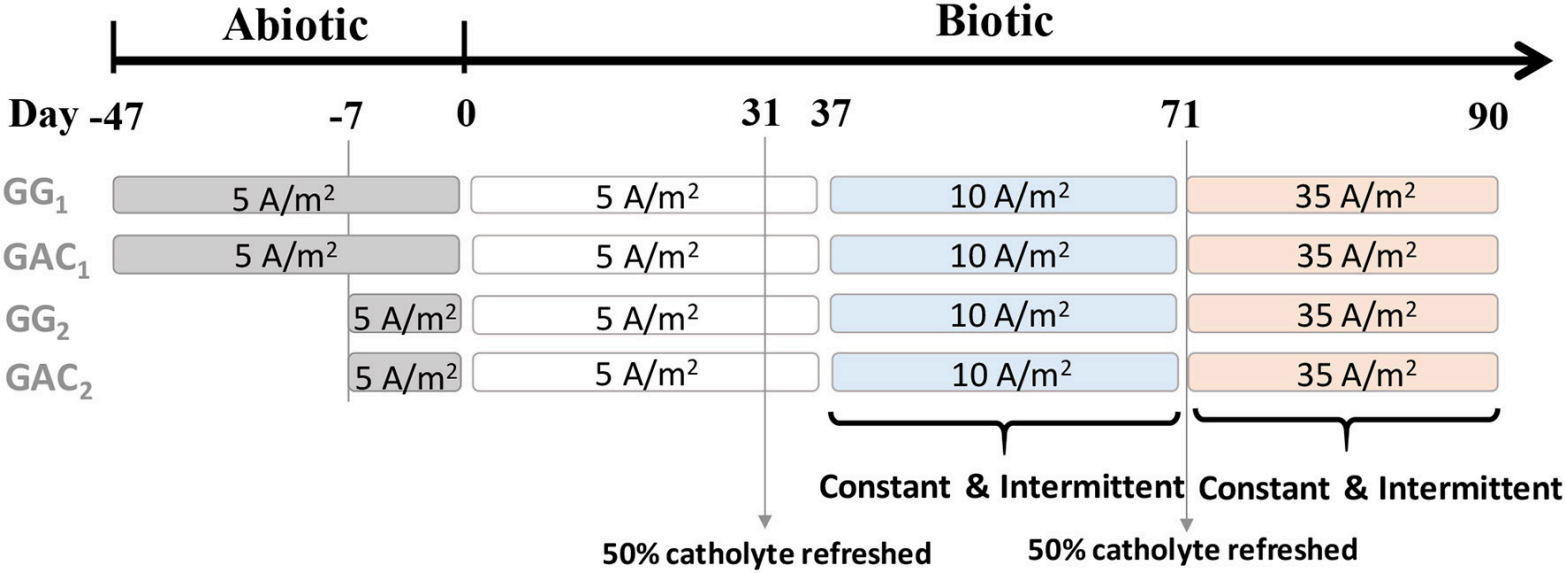
Overview of experimental conditions that were carried out for four methane-producing BES reactors. At day 0, all catholytes were inoculated. Days before inoculation are indicated as negative, days after inoculation are indicated as positive. During the phases of 10 and 35 A/m^2^cat_proj_, both constant and intermittent current supply modes were performed.

Experimental conditions are shown in Figure 1. All reactors were galvanostatically controlled (fixed current) by a potentiostat (Ivium n-Stat, Eindhoven, the Netherlands), which collected the cathode potential data from all reactors at intervals of 1 min. In this way, methane production rates can be regulated more directly than with cathode potential control, as the current determines the electrochemical reaction rate (Jörissen and Speiser, 2015). After inoculation, all reactors were operated at a fixed current of 5 A/m^2^cat_proj_ as startup period. The current of all reactors was increased from 5 to 10 A/m^2^cat_proj_ on day 37 and from 10 to 35 A/m^2^cat_proj_ on day 71. All cathodes were operated in batch. Half of the catholyte was replaced on days 31 and 70 to replenish buffer, nutrients and vitamins. The pH of each reactor was monitored daily by pH measurement of liquid samples (0.5 mL per sample) taken from anode and cathode chamber. All reactors were operated inside a temperature-controlled cabinet at 30°C.

For intermittent operation, a cycle time of 6 min (′) was used at three different current time-ON/time-OFF ratios: 4–2′, 3–3′, and 2–4′. Each ratio was tested for 20 h and was performed twice. After intermittent operation, all biocathodes were supplied with constant current for 20 h to investigate recovery after intermittent operation.

### Electrochemical analysis

Polarization curves were recorded before inoculation and on day 30 and day 90 after inoculation. For the polarization curve before inoculation, the cathode potential was controlled from −0.5 to −1.0 V with steps of 0.1 V; for the polarization curve after inoculation, the cathode potential was controlled from −0.1 to −0.7 V with steps of 0.05 V. Each potential step lasted 600 s for the GAC biocathodes, and 300 s for the GG biocathodes, as the latter required a shorter equilibrium time.

### Chemical analyses

The liquid and gas samples were taken from each reactor twice a week. Volatile fatty acids (VFAs), including formate, acetate and lactate, were determined in the liquid phase by high-performance liquid chromatography (HPLC) (Lindeboom et al., 2016), whereas the gas composition was measured by gas chromatography (GC) (Liu et al., 2016). The gas volume was quantified by emptying the gas bags with a syringe. The methane production rate was calculated and normalized to the projected surface area of the cathode (Equation 1) and the volume of the cathodic chamber (Equation 2), as follows:

(1) $$\begin{array}{r} \gamma_{CH4-A}=\frac{\text{V}\_\text{T}\times \text{C}\_\text{CH}4}{A_{proj}\times t} \end{array}$$

(2) $$\begin{array}{r} \gamma_{CH4-V}=\frac{V_{T}\times C_{CH4}}{V_{electrode}\times t} \end{array}$$

Here, γ_*CH*4−*A*_ (L CH_4_/m^2^ cat_proj_ /d) and γ_*CH*4−*V*_(L CH_4_/m^3^ cat /d) represent methane production rates; *V*_*T*_ (L) is the total volume by summing up the volume of the gas bag and the headspace (0.015 L); *C*_*C*_*H*__4__(%) is the methane fraction in the headspace; $A_{proj}\left(m^{2}\right)$ is the projected surface area of the graphite plate current collector and membrane; $V_{electrode}\left(m^{3}\right)$ is the cathodic chamber volume; t (d) is the experimental time between each headspace measurement (d).

### Current-to-methane efficiency

This indicates which percentage of the electrons ended up in the form of methane and is calculated as Equation 3.

(3) $$\begin{array}{r} \eta_{CH4}=\frac{\text{n}\_\text{CH}4{\times z}_{CH4}\times \text{F}}{\int_{\text{t }=\;0}^{\text{t}}\text{I dt}} \end{array}$$

F is the Faraday constant (96485 C/mol e^−^); n_CH4_ (mol) is total moles of CH_4_ produced; z_CH4_ is moles of electrons per mole of CH_4_ (8); I (A) is the current.

### Microbial community analysis

After operation at a current density of 35 A/m^2^cat_proj_, all reactors were disassembled inside an anaerobic chamber, and 0.5 g (wet weight) of the granules was taken from each cathode. In addition, 300 mL of the catholyte was taken from each reactor and filtered it over a 0.22 μm MF-MilliPore filter. Genomic DNA was extracted from each reactor samples with a Mo Bio PowerSoil DNA isolation kit for 0.5 g of the granular electrode and a Mo Bio PowerWater DNA isolation kit for the filter, according to the manufacturer's instructions. To investigate both bacteria and archaea, firstly amplification of 16S rRNA gene fragments was carried out by using a two-step PCR protocol, and then DNA was quantified using a Qubit^®;^ dsDNA BR Assay Kit and a DeNovix DS-11 FX spectrophotometer/fluorometer (DENovix Inc., Wilmington, DE, USA), finally the 16S rRNA gene Miseq sequencing data were analyzed using Galaxy/NG-Tax, an in-house pipeline (see detailed information in Supportive Information, under B). Bray-Curtis similarities were calculated between reactors (biocathodes and used catholytes) from the microbial community relative abundance data with Primer-E software, version 7 (LaBarge et al., 2017).

## Results and discussion

### High methane production rates directly linked to current density

**Figure 2:**
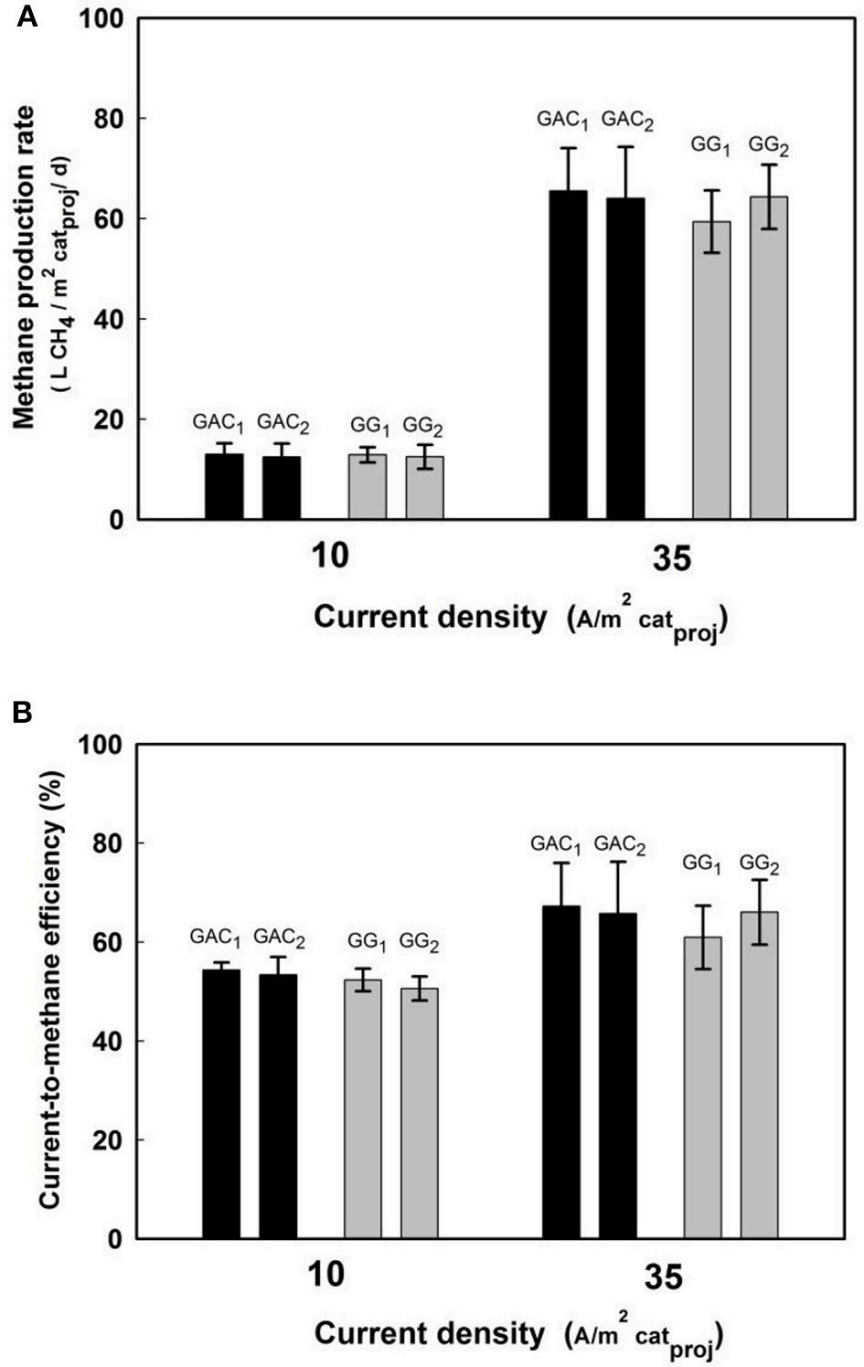
Methane production rates **(A)** and current-to-methane efficiencies **(B)** at current densities of 10 A/m^2^cat_proj_ and 35 A/m^2^cat_proj_ for GAC and GG biocathodes. Data were collected throughout a period of 2 weeks with stable performance for every reactor. Shown are the average value and standard deviation of four samples for each reactor and current density.

We determined methane production rates at two different current densities of 10 and 35 A/m^2^cat_proj_. At the current density of 35 A/m^2^cat_proj_, the methane production rates were around 65 L CH_4_ /m^2^cat_porj_/d for both cathode materials (Figure 2A). As methane production rates were directly related to current density, they were almost four times higher than at 10 A/m^2^cat_proj_. The current-to-methane efficiencies for the GAC and GG reactors (Figure 2B increased from 55% at 10 A/m^2^cat_proj_ to 67% at 35 A/m^2^cat_proj_. No H_2_ and volatile fatty acids were detected in any of the reactors at these two current densities, which suggests that during the stable performance period, the methanogenic activity was high enough to utilize these components if they were produced. Possible other electron sinks are biomass growth (Geppert et al., 2016), or loss of methane via membrane, tubes, and connections within the reactor (Skovsgaard and Jacobsen, 2017), especially considering our relatively long sampling intervals (3–4 days). Also, reduction of oxygen generated at the anode could play a role (Van Eerten-Jansen et al., 2012). The methane production rates achieved with GAC and GG at constant current in this study were compared with similar carbon-based electrodes in other studies (Geppert et al., 2016; Table 1). One the one hand, methane production rate at 35 A/m^2^ were several times higher than those in other studies. On the other hand, methane production rate at 10 was similar with other studies, but it is interesting to note that the cathode potentials of GAC biocathodes were quite different in this case, −0.55 V for GAC biocathodes compared to −1.1 V for biocathodes, i.e., Villano et al.

**Table 1:** Comparison of methane production rates for similar 3D carbon-based electrodes in methane-producing BESs when water was used as electron donor.

**Electrode material**	**Current density**		**Methane production rate**		**Current-to-CH_4_ Efficiency (%)**	**Cathode potential (V vs. Ag/AgCl)**	**Reference**
	**(A/m^2^cat_proj_)**	**(kA/m^3^cat)**	**(LCH_4_/m^2^cat_proj_/d)**	**(m^3^CH_4_/m^3^cat/d)**			
GAC	10	0.67	15	1.0	54	-0.52	This study
GAC	35	2.3	65	4.3	66	-0.58	This study
GF	0.21	0.070	0.13	0.045	23	-0.75	Van Eerten-Jansen et al., 2012
GF	2.9	0.97	5.1	1.7	73	-0.9	van Eerten-Jansen et al., 2015
GF	7.1	2.5	8.8	3.1	69	-1.3	Liu et al., 2017
GG	10	0.67	15	0.97	52	-0.9	This study
GG	35	2.3	62	4.1	67	-1.1	This study
GG	3.8	0.13	17	0.56	79	-1.1	Villano et al., 2013

### Methane production was related to total charge also in intermittent mode

After all the biocathodes achieved a stable methane production rates at a constant current supply of 10 A/m^2^cat_proj_, intermittent current (at the same current density) was supplied to all biocathodes with three different time intervals: 4–2′, 3–3′, and 2–4′. Methane production rate of each biocathode is shown in **Figure 4A**, calculations based on the 20 h period for each time interval. Higher current time-ON/time-OFF interval supplied to the biocathodes resulted in higher methane production rates, with 9.5 L CH_4_/m^2^ cat_proj_/d at 4–2′, 5.5 L CH_4_/m^2^ cat_proj_/d at 3–3′ and 4.0 L CH_4_/m^2^ cat_proj_/d at 2–4′, meaning that charge provided during ON-time was used to generate methane.

**Figure 3:**
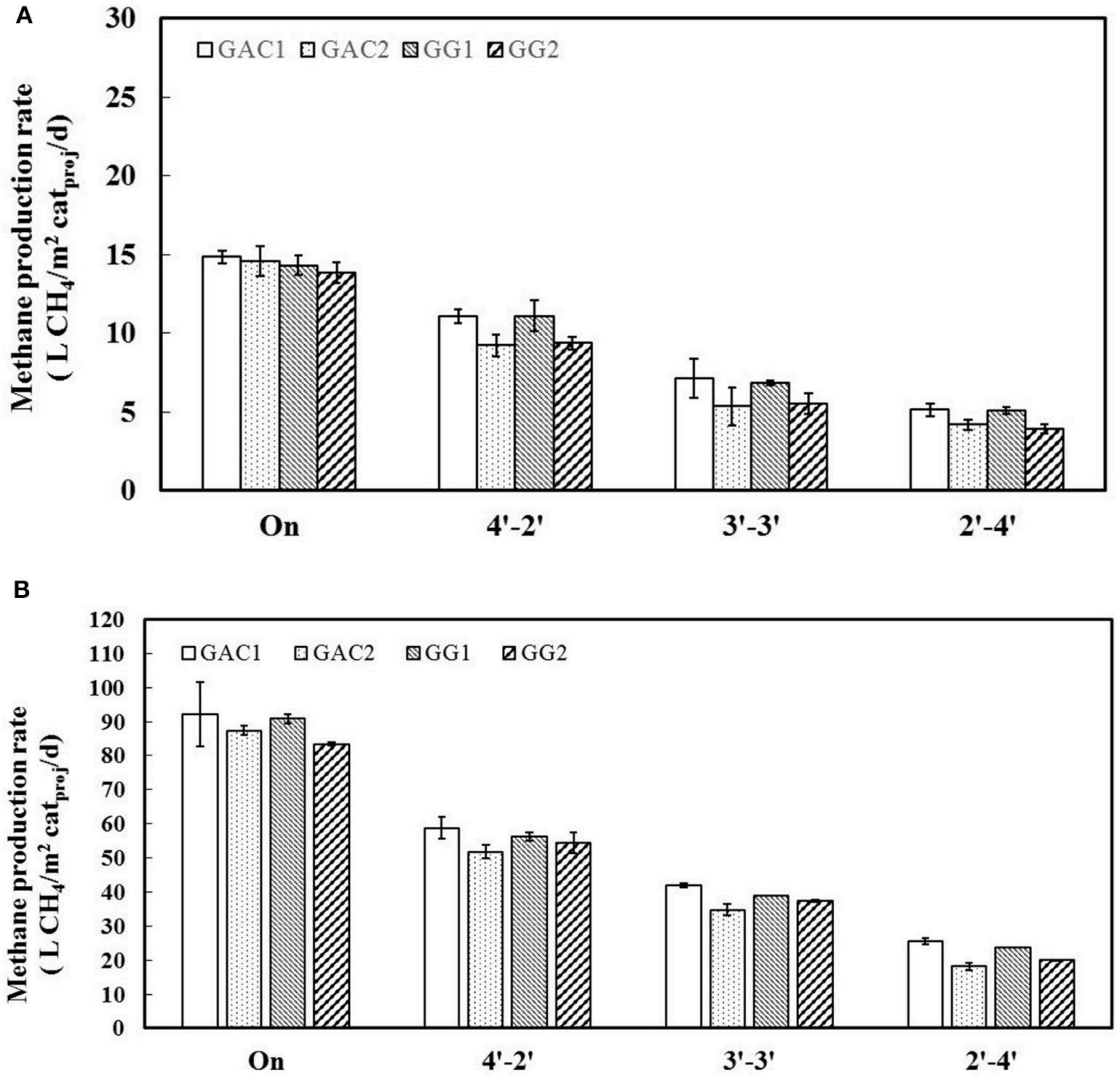
Methane production rates for all biocathodes when they were supplied with constant current and intermittent current. Three different current time-ON/time-OFF intervals (4–2′, 3–3′, and 2–4′) were carried out. The current density during the current time-ON was 10 A/m^2^cat_proj_ **(A)** and 35 A/m^2^cat_proj_ **(B)**. For each operational condition, duplicate experiments were performed. The standard deviations are shown as an error bar, whereas the average value is shown as a column.

When the current density was increased from 10 to 35 A/m^2^cat_proj_, the methane production rate at continuous current supply increased from 15 L CH_4_/m^2^ cat_proj_/d at 10 A/m^2^ (Figure 3A) to 90 L CH_4_/m^2^ cat_proj_/d at 35 A/m^2^ (Figure 3B). Again, an increase in methane production rate was observed along with increasing time-ON/time-OFF ratios. Moreover, we compared our experimental data with the theoretical methane production calculated for the different current time-ON/time-OFF ratios (Figure S4 in Supporting Information, under D). The close fit between measured and calculated data shows that methane generation is closely linked to the charge provided to the biocathode, for both GG and GAC.

### Intermittent current operation does not influence biocathode activity

**Figure 4:**
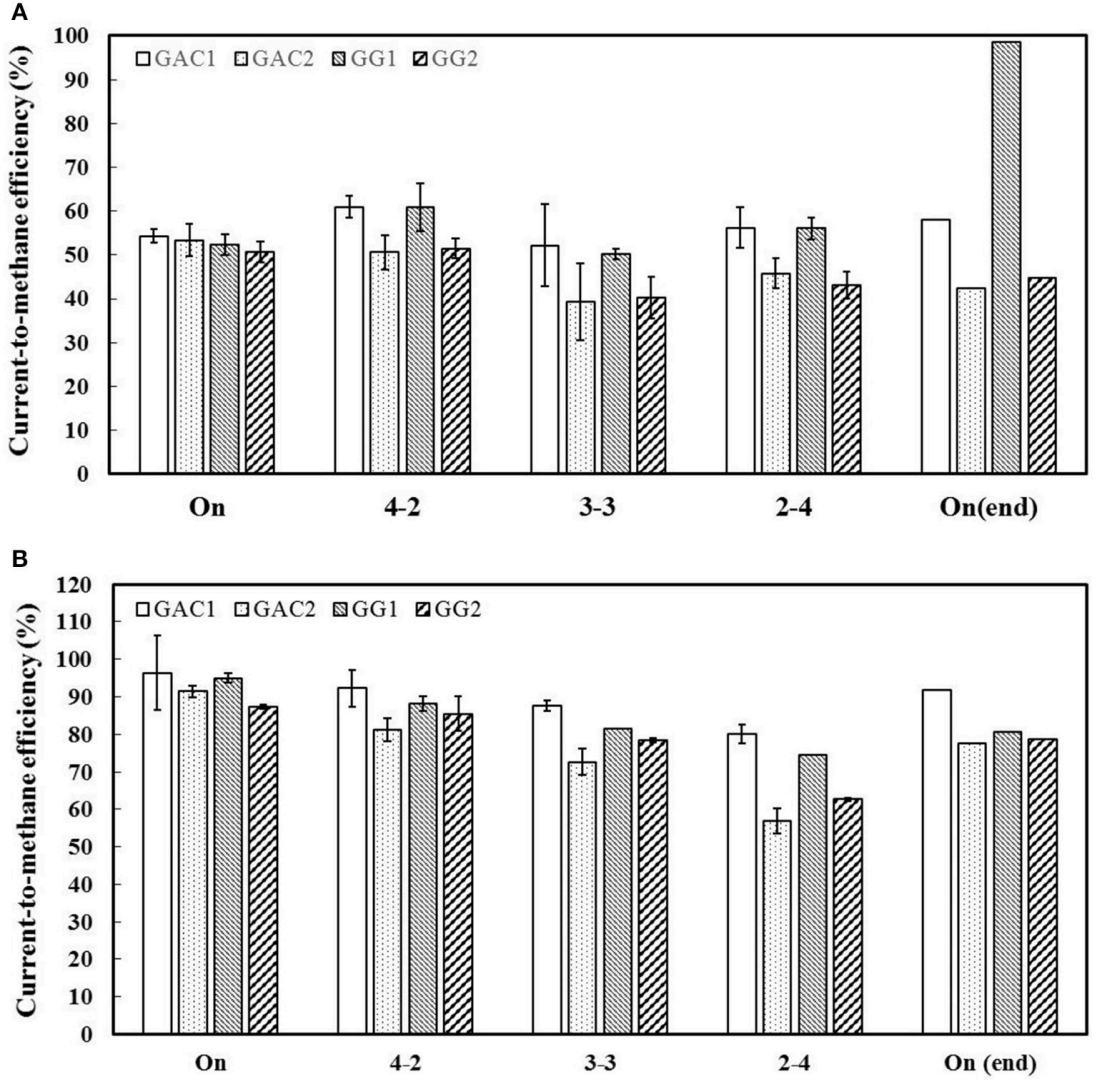
Current-to-methane efficiencies (%) for all reactors when they were supplied with constant and intermittent current. Three different current time-ON/time-OFF ratios (4–2′, 3–3′ and 2–4′) were carried out. The current density during the current time-ON was 10 A/m^2^cat_proj_ **(A)** and 35 A/m^2^cat_proj_ **(B)**. For each operational condition, duplicate operations were performed. The maximum and minimum values are shown as an error bar, whereas the average value is shown as a column.

Studies on bioanodes have shown that GAC can store charge in the electric double layer when used in Microbial Fuel Cells (Deeke et al., 2015; Lu et al., 2015), whereas GG with low capacitance does not show this charge storage behavior. This higher capacitance of GAC biocathodes was expected to result in smaller fluctuations in cathode potential, and as a possible electron buffer, compared to GG biocathodes during intermittent operation (Borsje et al., 2016). As shown in Figure S5, the cathode potentials of GAC biocathodes during intermittent current indeed kept stable around −0.5 V, whereas the cathode potentials of GG biocathodes changed in the range from −0.6 to −1.0 V. These results might indicate that intermittent current operation would affect GAC biocathodes less than GG biocathodes. However, at a current density of 10 A/m^2^cat_proj_, all biocathodes, operated under different current time-ON/time-OFF intervals, had a similar current-to-methane efficiency of 50–60% (Figure 4A). When the current density was increased from 10 to 35 A/m^2^cat_proj_, the current-to-methane efficiency was also constant with a slight decrease along with the longer time-OFF intervals (Figure 4B). After these intermittent operations, an additional constant current supply for 20 h was operated for all biocathodes to verify if the initial activity was restored after intermittent operation. As shown in Figure 4, the current-to-methane efficiencies of all biocathodes after intermittent operations were similar to those at constant current supply, indicating that biocathodes were not affected by the intermittent operation at these two current densities of 10 and 35 A/m^2^cat_proj_, for both materials. In addition, the methane production rate of all biocathodes in our study kept stable, even though all biocathodes had experienced around 60 min of open circuit period during each headspace and pH sampling time. Our results are, however, different from those results found in the previous study that the methane production rate decreased by 87% after an open circuit period of 45 min, and it took 4 months before performance was back at the original level (Bretschger et al., 2015). The discrepancy could be due to that the quantity and/or bioactivity of biofilm growth on our granular carbon-based electrodes is higher than those on the carbon cloth electrode used in that study, as higher current density was found on our biocathodes (5 A/m^2^) compared with their biocathodes (0.06 A/m^2^). To conclude, current-to-methane efficiency (%) remained quite stable under the different current supply modes at 10 A/m^2^, and showed a slight decrease with increasing OFF-time at 35 A/m^2^.

**Figure 5:**
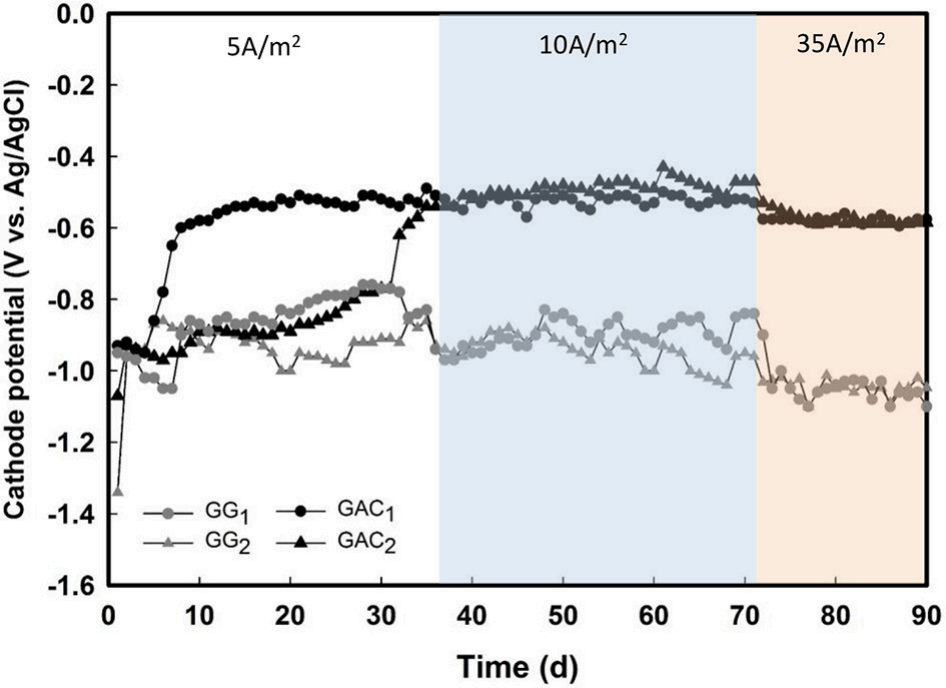
Average daily cathode potentials of all the reactors after inoculation. Both GAC biocathodes showed a steep increase in cathode potential, whereas the cathode potential for both GG biocathodes remained constant, and decreased with increased current density.

It is worth notifying that current-to-methane efficiencies obtained at continuous current density of 35 A/m^2^cat_proj_ in Figure 4B, are even higher than those achieved at the same current density reported in Figure 2B. This discrepancy could be due to the different durations between headspace sampling: 20 h for Figure 5 and 3–4 days for Figure 3. Shorter duration between headspace sampling could mitigate losses via H_2_ or O_2_ leakage from the joints of the experimental set-up.

### Biocathodes with granular activated carbon produced methane at low overpotentials

Although there is no difference between GAC and GG biocathodes during constant and intermittent operation in terms of methane production rate (Figures 2A, 3) and current-to-methane efficiency (Figures 2B, 4). Interestingly, the cathode potentials of the GAC biocathodes were different from the GG biocathodes.

Directly after inoculation, all the reactors had similar cathode potentials of about −0.90 V (Figure 5). The cathode potential of GAC_1_ changed from −0.90 to −0.52 V between day 7 and 10, whereas the cathode potential of GAC_2_ changed from −0.80 to −0.52 V between day 30 and 37. The cathode potentials of the GG reactors remained stable around −0.92 V long after inoculation and became slightly more negative around day 37 and day 70 due to the increases in current density. These potential differences between GAC and GG biocathodes were also seen in the polarization curves at day 30 (Figure S2B in the Supporting Information, under C) and day 90 (Figure S2C in the Supporting Information, under C). These polarization curves show that the onset of current for GAC biocathodes occurred at a more positive potential from −0.5 V to even −0.4 V, whereas the current densities of GG biocathodes were negligible in the whole range of cathode potentials tested (−0.7 to −0.3 V). For the current obtained in GAC at −0.5 V during the polarization experiment, other possible secondary reactions, e.g., hydrogen, acetate or formate, could play a role. As with these intermediates, quick consumption by methanogens could lead to undetectable levels of these intermediates. Nevertheless, the onset potentials of the bare GAC electrodes (Figure S2A in the Supporting Information, under C) were around −0.7 V, the difference indicating the catalytic effect of the cathodic microorganisms growth on the GAC electrodes.

To exclude that the measurement of cathode potential was influenced by the fact that the reference electrode was placed outside the cathode compartment, we inserted a new Ag/AgCl reference electrode into one of GAC cathodic chamber as close as possible to the granular bed. The cathode potential was around −0.43 V, which was 100 mV less negative than the cathode potential (−0.52 V) measured outside the cathode chamber, pointing out that the actual cathode potential was even less negative than that was measured. To our knowledge, these cathode potentials for GAC are the least negative ones (i.e., lowest overpotential) reported in the literature for methane-producing BESs (Geppert et al., 2016). It is likely that methane production at a cathode potential of −0.52 V has not been reported before due to the fact that all methane-producing biocathodes in other studies were operated at a constant potential rather than at a constant current. Actually, most of the studies have used cathode potentials more negative than −0.7 V vs. Ag/AgCl to supply a sufficiently high overpotential for methane generation (Siegert et al., 2014; Villano et al., 2016; LaBarge et al., 2017). Switching from galvanostatic control to potentiostatic control with an active biocathode resulted in similar rates and efficiencies (results are shown as Figure S6 in the Supporting Information, under F). Galvanostatic control is thus useful to achieve methane production at low overpotential, but can be changed to potentiostatic control once an active biocathode is present, without loss in activity.

At this point, it is unclear why the cathode potential of GAC changed to −0.52 V, while cathode potential of GG remained at −0.90 V. The high specific surface area and average smaller size of GAC (764 m^2^/g, 1–3 mm) relative to GG (0.438 m^2^/g, 3–5 mm) may have played a role, but does not explain the mechanism of methane formation. It is worth notifying that similar phenomenon had been shown in a previous study where the presence of GAC in anaerobic digestion stimulated methane production rate, whereas graphite electrode did not affect the performance, for reasons not yet understood (Dang et al., 2016). In our study, the cathode potential of −0.52 V is 0.1 V more positive than the thermodynamic equilibrium potential for hydrogen evolution (−0.62 V) under the biological conditions (*T* = 30°C, *P* = 1 bar, pH = 7) (Beese-Vasbender et al., 2015). Such less negative cathode potential and the undetectable hydrogen in GAC biocathodes suggests that the change in potential for methane production on GAC biocathodes observed here could be related to direct electron transfer. However, hydrogen as an intermediate for methane production at GAC biocathode cannot be excluded as the local hydrogen pressures and local pH values are not known. Additional research is needed to measure the actual values of local pH and hydrogen pressure on the biocathode by using microsensor, and therefore, providing insight into the relationship between mechanisms of electron transfer and different conductive materials.

The changes in the biocathode potentials of the GAC reactors occurred on different days (Figure 5). The reason for that may be that in GAC_1_, which had been operated and adjusted during 2 months before inoculation to perform electrochemical measurements, the catholyte and/or electrode may already have contained methanogens before inoculation. Indeed, a minor amount of CH_4_ was already detected in the headspace of GAC_1_ during the phase before inoculation (data not shown). The fluctuations of the cathode potentials, especially at current densities of 5 A/m^2^cat_proj_ and 10 A/m^2^cat_proj_, were probably the result of fluctuations in catholyte pH due to intermittent CO_2_ supply (Jourdin et al., 2015). After changing to continuous CO_2_ supply and a current density of 35 A/m^2^cat_proj_ on day 71, the pH of the catholyte and the cathode potentials remained more stable (Figure S3, supporting information, under C).

### Microbial community analysis revealed *Methanobacterium* as dominant species

**Figure 6:**
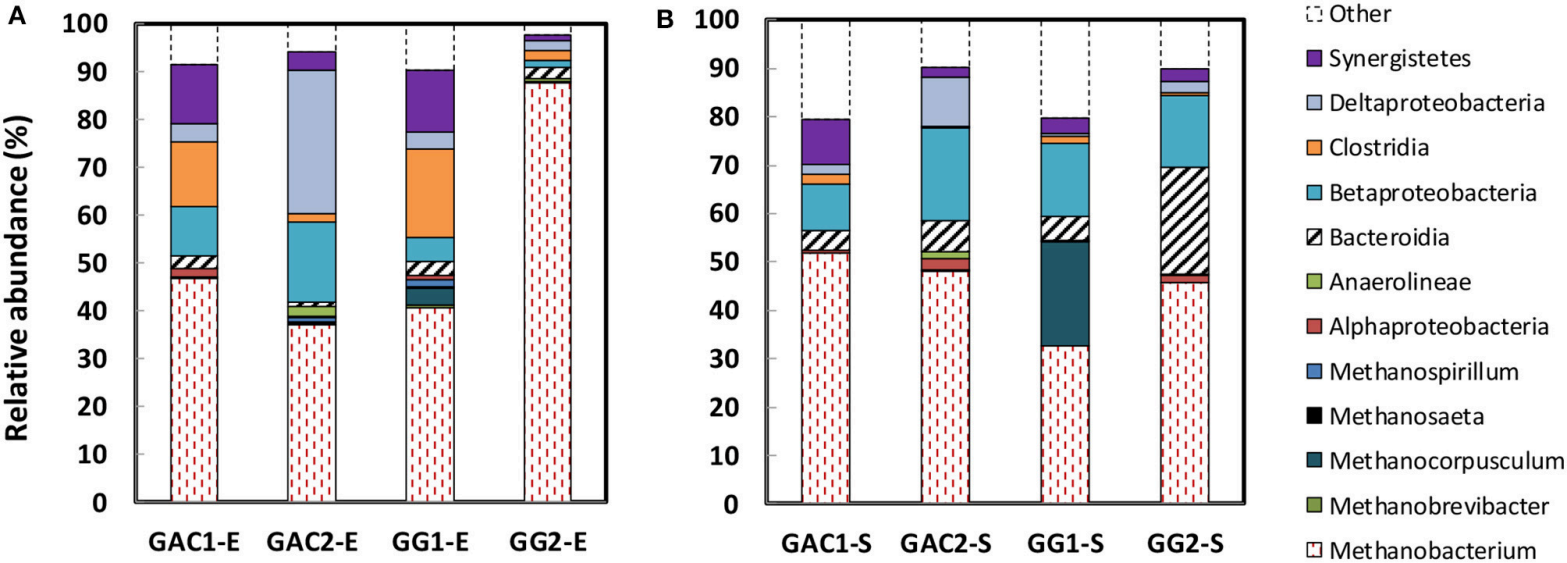
Taxonomic distribution of microbial populations with >2% relative abundance by 16S rRNA gene sequences. Samples from all four reactors were taken from: **(A)** biofilm on granular biocathodes (GAC_1_-E, GAC_2_-E, GG_1_-E, GG_2_-E); **(B)** suspended cells within the catholyte (GAC_1_-S, GAC_2_-S, GG_1_-S, GG_2_-S).

Microbial communities of biofilm and catholyte were characterized for all reactors to investigate whether different microbial communities developed on the two cathode materials. Table S1 in the Supporting Information shows the community similarity results for all granules. All cathodic communities (both in biofilm and catholyte) were dominated by hydrogenotrophic methanogens (*Methanobacterium*), which has been found in many other studies (Van Eerten-Jansen et al., 2013; Cai et al., 2016; LaBarge et al., 2017) regardless of electrode material and inoculum source (Figure 6). Another hydrogenotrophic methanogen, namely *Mthanocorposculum*, was detected 21% in the catholyte of GG_1_. The GAC electrode samples showed a greater relative abundance of Proteobacteria (*Deltaproteobacteria and Betaproteobacteria*) with 14% for GAC_1_ and 47% for GAC_2_, relative to 8.7% for GG_1_ and 3.4% for GG_2_, As exoelectrogens like *Geobacter* sp. belong to the proteobacteria, the most common phylum of bacteria found on the anode of microbial fuel cells (Hasany et al., 2016), this may be related to the lower overpotentials measured for GAC.

## Conclusion

In this paper, we have shown that both GAC and GG are suitable cathode materials for high methane production rates in methane-producing BESs. Intermittent current operation resulted in stable methane production for both materials, and original performance was restored directly after intermittent operation. GAC biocathodes showed lower overpotentials than GG; the mechanism behind this needs to be further investigated. Granular biocathodes thus hold promise for the practical application of methane-producing BESs for renewable electricity storage.

## Author contributions

AtH conceived the original idea and together with CB supervised the project. DL and MR-P carried out the experiment. DL wrote the manuscript with support from MR-P, SN. LC-J, and FG, helped supervise the project. SN helped conducted the microbial community analysis.

### Conflict of interest statement

The authors declare that the research was conducted in the absence of any commercial or financial relationships that could be construed as a potential conflict of interest.
