# Supplementary material for: Granular Carbon-Based Electrodes as Cathodes in Methane-Producing Bioelectrochemical Systems
Source: Front Bioeng Biotechnol. 2018 Jun 12;6:78. doi: 10.3389/fbioe.2018.00078 (PMC6005836; doi:10.3389/fbioe.2018.00078)
Supplement: Supplementary file 1 [file Data_Sheet_1.docx]

SUPPORTING INFORMATION (12 pages)

**Granular Carbon-based Electrode as Cathode Material in Methane-Producing Bioelectrochemical Systems**

Dandan Liu*, Marta Roca-Puigros*, Florian Geppert***, Leire Caizán-Juanarena*, Susakul Palakawong Na Ayudthaya**, Cees Buisman*, Annemiek ter Heijne*

* Sub-Department of Environmental Technology, Wageningen University& Research, Bornse Weilanden 9, 6708 WG Wageningen, The Netherlands

**Laboratory of Microbiology, Wageningen University & Research, Bornse Weilanden 9, 6708 WG Wageningen, The Netherlands

*** Fraunhofer Institute for Environmental, Safety, and Energy Technology UMSICHT, Osterfelder Strasse, 3, 46047 Oberhausen, Germany

**Corresponding Author: Annemiek ter Heijne (E-mail:** [**annemiek.terheijne@wur.nl**](mailto:annemiek.terheijne@wur.nl)**)**

**12 pages, 6 Figures, 2 Tables**

**A. Methane-producing BES setup**

**(a)**


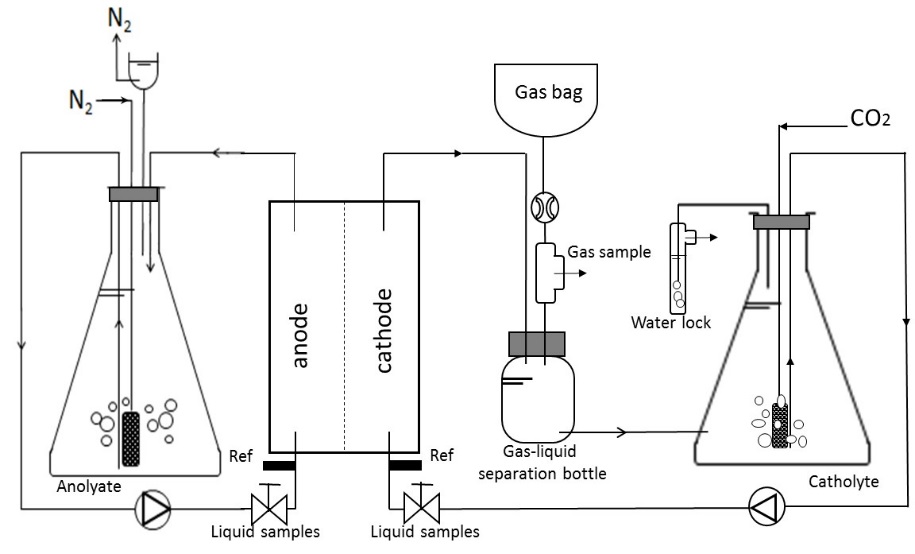


**(b)**

**6**

**2**

**3**

**Biocathode**

**7**

**Anode**

**8**

**5**

**Potentiostat**

**1**

**4**

**3**

Figure S1. Schematic overview of the experimental set-up (a) and Schematic depiction of the methane-producing BES (b). Each reactor contained an anodic and a cathodic chamber. The anode was a titanium plate covered with platinum-iridium (1). The anode chamber was filled with glass beads of a 7-mm diameter (Hecht-Assistent, Sondheim v. d. Rhön, Germany) (2) and a plastic spacer (Sefar Nitex 06-3300/59, Buffalo, NY, USA) (3) to protect the cation exchange membrane (4). The cathode chamber was completely filled with the cathode, GG or GAC (5), together with a plastic spacer (3). The electrons were supplied to the bed of granules from the external circuit via a graphite plate (working area of 22 cm^2^) (6). Both chambers had an inlet (7) and an outlet (8) with anolyte and catholyte flowing through the anodic and cathodic chamber, respectively.

**B. 16S rRNA Miseq Sequencing Analysis**

Extracted DNA from selected samples was kept at -20 ºC for bacterial and archaeal community analyses. DNA was measured with a DeNovix DS-11 FX spectrophotometer/fluorometer (DENovix Inc., Wilmington, DE, USA) and the DNA concentrations (ca. 20 ng/µl) were used as PCR templates.

The amplification of bacterial and archaeal 16S rRNA gene fragments was performed using a two-step PCR protocol. For the bacterial gene fragments, first PCR was done with universal primers 515f and 806r (Parada et al. 2015), while for the archaeal gene fragments universal primers 518f(Wang and Qian 2009) and 905r(Kvist et al. 2007) were used. The first PCR for both bacterial and archaeal 16S rRNA gene fragments was performed in a total volume of 50 µl, containing 2.5 µl of each forward and reverse primer, 0.5 µl (2 unit) of the DNA polymerase, 10 µl of 5 x HF-buffer, 1 µl (200 µM) dNTP mix, 1 µl of DNA template, and 32.5 µl of nuclease-free sterile water. The PCR program for bacterial amplification was as follows: a pre-denaturing step at 98 ºC for 3 min, followed by 25 cycles at 98 ºC for 10 s, 50 ºC for 20 s, 72 ºC for 20 s, and a post-elongation step of 10 min at 72 ºC. For archaeal amplification, the PCR program was as follows: a pre-denaturing step at 98 ºC for 30 s, followed by 25 cycles at 98 ºC for 10 s, 60 ºC for 20 s, 72 ºC for 20 s, and a post-elongation step of 10 min at 72 ºC. PCR amplifications were carried out in technical duplicates.

After positive amplification, the second PCR for both bacterial and archaeal 16S rRNA gene fragments was separately done using the same protocol in 100 µl, containing 10 µl of the bar-coded primer mix, 1 µl (2 units) of the DNA polymerase, 20 µl of 5 x HF-buffer, 2 µl (200 µM) dNTP mix, 5 µl of DNA template, and 62 µl of nuclease free sterile water. The second PCR for both the bacterial and the archaeal gene fragments was carried out with eight-base specific barcodes as previously described(Hamady et al. 2008), using Phusion Hot Start II High-Fidelity DNA polymerase (Thermo Fisher Scientific, Waltham, MA, USA). PCR amplification was performed using a G-Storm cycler (G-storm, Essex, UK). The second PCR program was as follows: a pre-denaturing step at 98 ºC for 30 s, followed by 5 cycles at 98 ºC for 10 s, 52 ºC for 20 s, 72 ºC for 20 s, and a post-elongation step of 10 min at 72 ºC. Bar-coded PCR products were checked for positive amplification on an agarose gel and then were purified using CleanPCR kit system according to the manufacturer’s instructions (CleanNA, Alphen aan den Rijn, the Netherlands).

DNA was quantified using a Qubit® dsDNA BR Assay Kit and a DeNovix DS-11 FX spectrophotometer/fluorometer (DENovix Inc., Wilmington, DE, USA). All samples were pooled in equimolar amounts (200 ng of DNA per sample) to create a library, which was then purified again with the CleanPCR kit to a final volume of 35 µl. The library was dispatched for paired-end Illumina MiSeq sequencing at GATC Biotech (Konstanz, Germany).

16S rRNA gene Miseq sequencing data were analyzed using Galaxy/NG-Tax, an in-house pipeline, as previously described by Ramiro-Garcia et al. (Ramiro-Garcia et al. 2016). Paired-end libraries were filtered to obtain only read pairs with perfectly matching barcodes and those barcodes were then used to detach reads from the sample. The Silva 16S rRNA gene reference database (release 128) was used for the taxonomic classification(Quast et al. 2012).

**Bray-Curtis Similarities**

The Bray-Curtis similarity is a useful coefficient to measure the resemblance between samples containing multivariate data(Somerfield 2008). Table S1 shows the Bray-Curtis similarities for the methane-producing BES reactors in our study.

Table S1. Bray-Curtis similarities (%) between electrodes samples (E) and catholyte samples (S), taken from all the reactors after the operation at a current density of 35 A/m^2^cat_proj_. Darker shades indicate greater resemblance between samples.

|  | GAC1-E | GAC2-E | GG1-E | GG2-E | GAC1-S | GAC2-S | GG1-S | GG2-S |
| --- | --- | --- | --- | --- | --- | --- | --- | --- |
| GAC1-E |  | **64** | **87** | **58** | **81** | **76** | **60** | **74** |
| GAC2-E | **64** |  | **59** | **47** | **60** | **75** | **60** | **65** |
| GG1-E | **87** | **59** |  | **52** | **76** | **65** | **60** | **65** |
| GG2-E | **58** | **47** | **52** |  | **63** | **58** | **42** | **56** |
| GAC1-S | **81** | **60** | **76** | **63** |  | **76** | **72** | **75** |
| GAC2-S | **76** | **75** | **65** | **58** | **76** |  | **66** | **83** |
| GG1-S | **60** | **60** | **60** | **42** | **72** | **66** |  | **66** |
| GG2-S | **74** | **65** | **65** | **56** | **75** | **83** | **66** |  |

**C. Additional Information on Performance**

(a) (b)

**
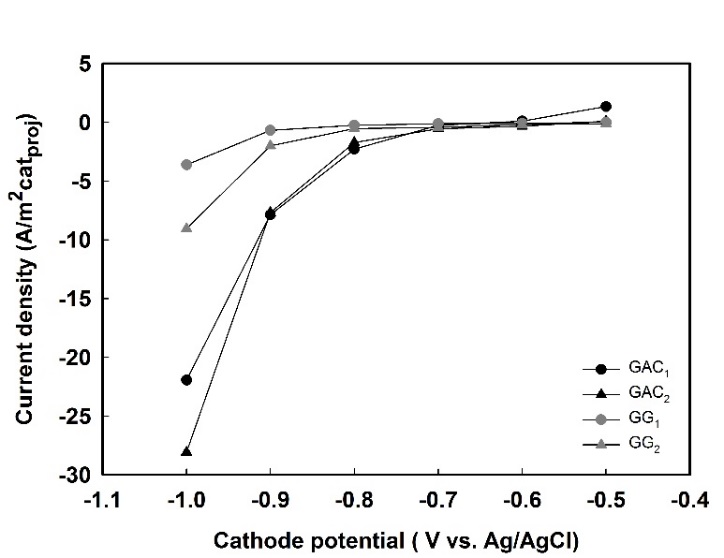

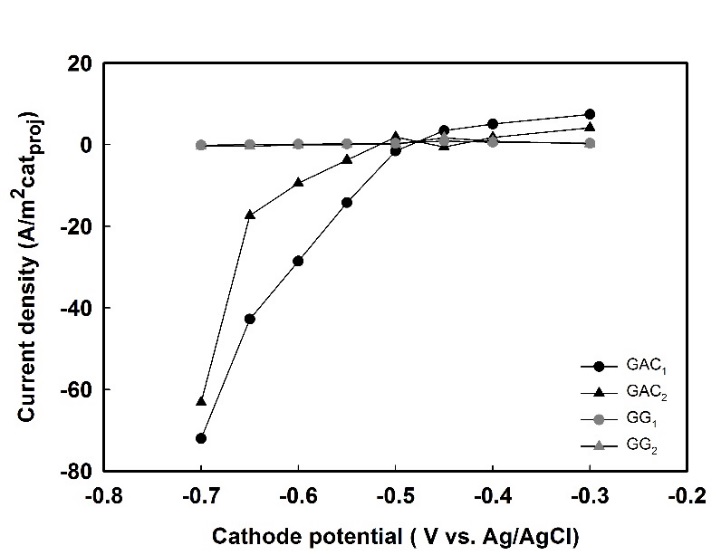
**

(c)


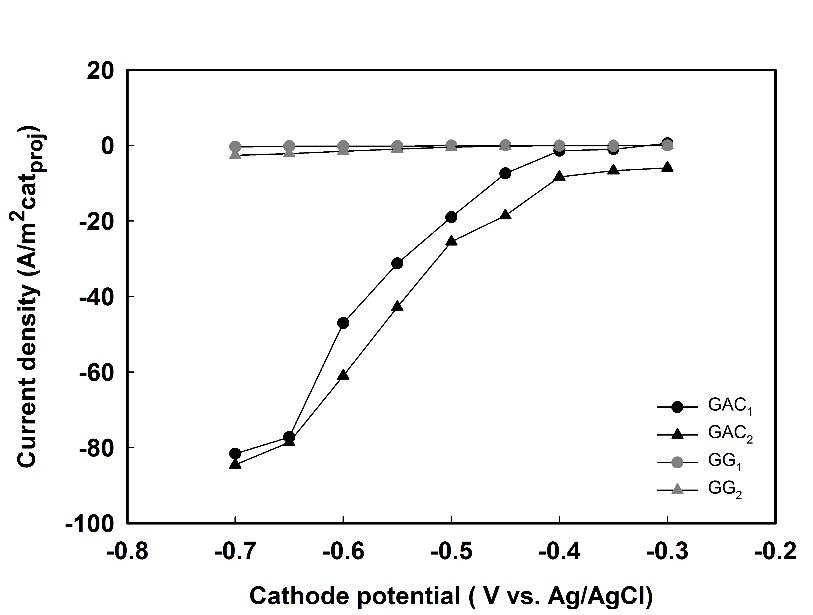


Figure S2. Polarization curves acquired before inoculation (a), after inoculation on day 30 (b) and day 90 (c) for all reactors**.** For bare electrode materials (a), GAC had a less negative onset potential (about -0.7 V vs. Ag/AgCl) than GG (about -0.8 V vs. Ag/AgCl). In the presence of the catalytic effect of microorganisms on electrodes (Figures S2b and S2c), the onset potentials of the GAC biocathodes became more positive during operation. The current densities reached values that are in line with the results obtained during galvanostatically controlled operation.


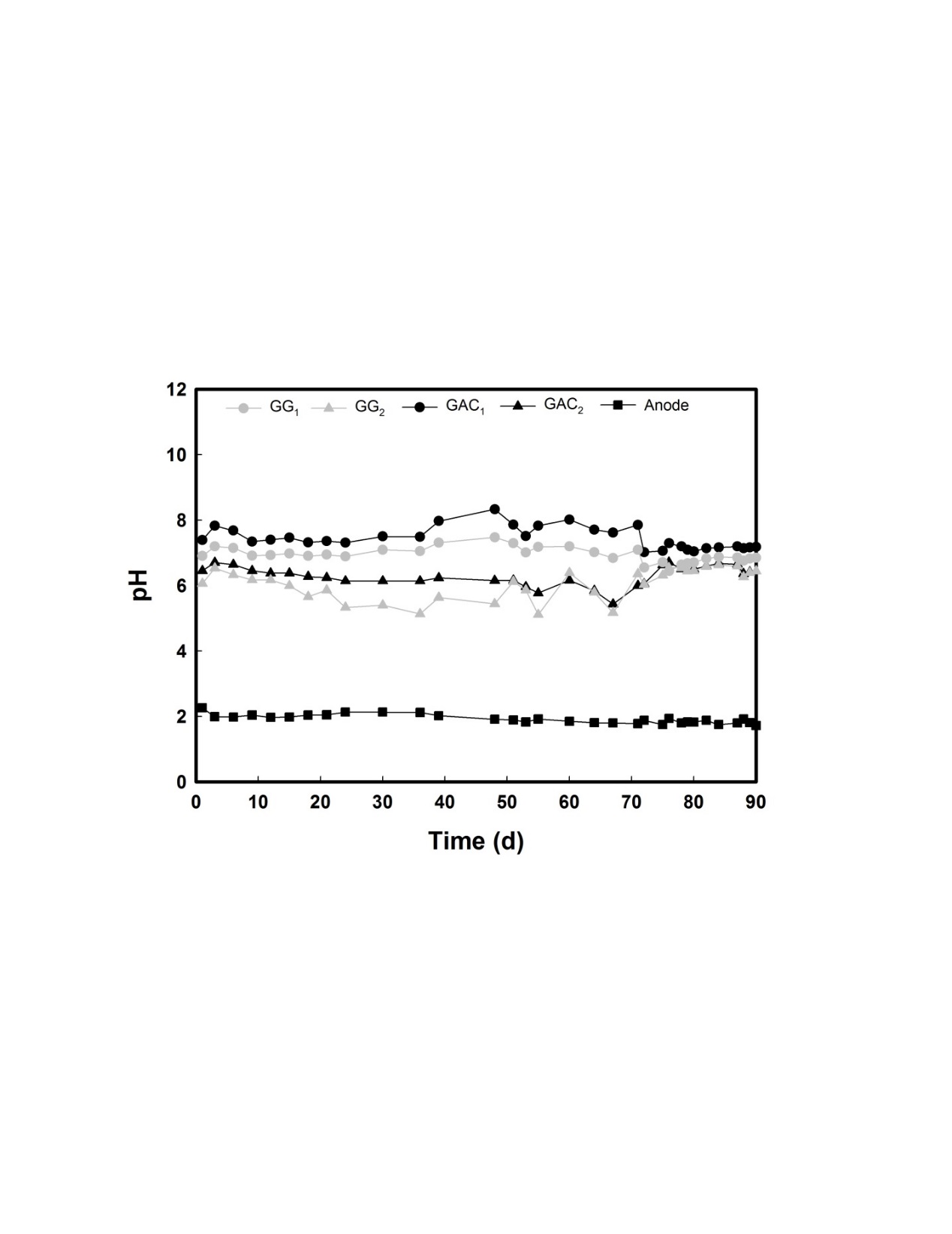


Figure S3. pH values for anolyte and catholyte in all reactors after inoculation. Catholyte pH of all reactors fluctuated between 6 and 8 during the period with intermittent CO_2_ supply, but became stable at 7 during the period with constant CO_2_ supply. All reactors shared the same anolyte with a stable pH of 2 throughout the whole experiment.

**D**. **Comparision of methane production rates between experimental data and theoretical results.**

Figure S4. Overview of methane production rates of four biocathodes: GAC_1_ (a), GAC_2_ (b), GG_1_ (c) and GG_2_ (d), at different current supply modes: constant current (On) and intermittent current. Three different current time-ON/time-OFF ratios (4 - 2, 3 - 3 and 2 - 4) were carried out. The methane production rates of experimental data are shown as filled circles, while the methane production rates of theoretical data calculated according to different current time-On/time-OFF ratios are decapitated as a solid line. The calculation assumed that the methane production rate at the constant current supply of 35 A/m^2^cat_proj_ was same for both experimental data and theoretical data. The theoretical data meet the experimental data, which indicates a linear relationship between the amount of methane produced in biocathodes and the current time-ON/time-OFF ratios.

**E. Cathode potentials of GAC and GG biocathodes during intermittent current supply.**

1. (b)


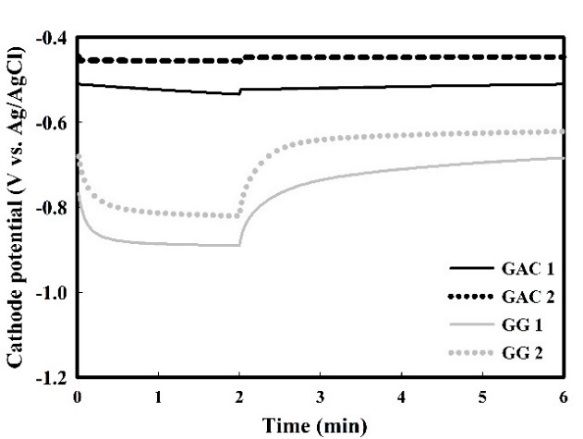


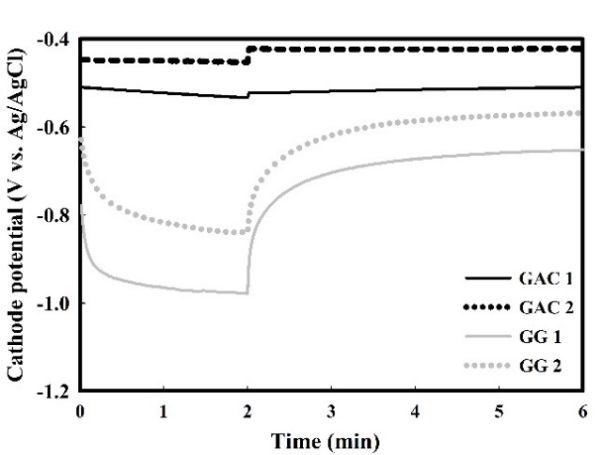


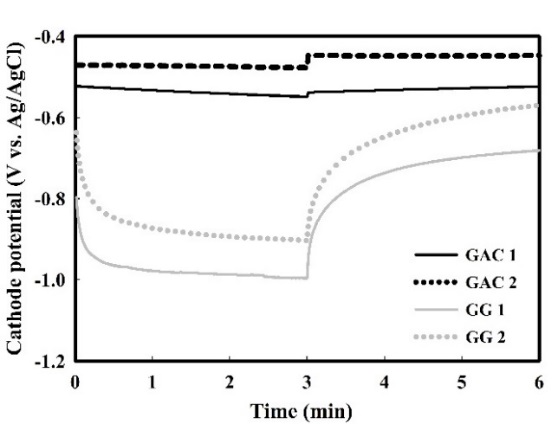

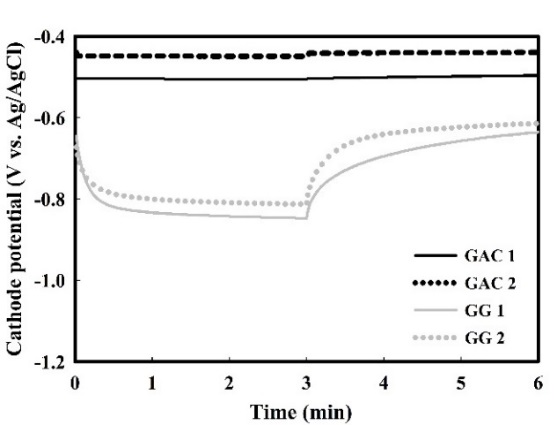
 (c) (d)


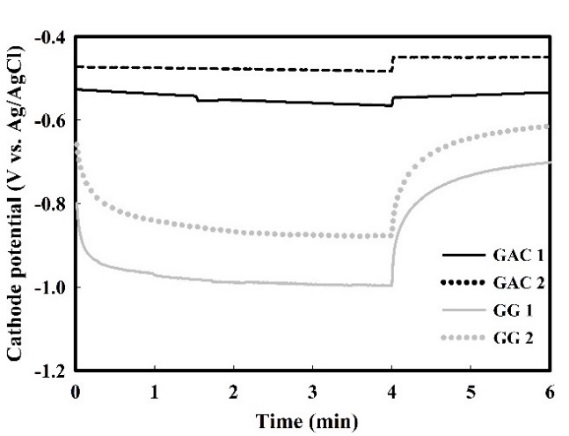

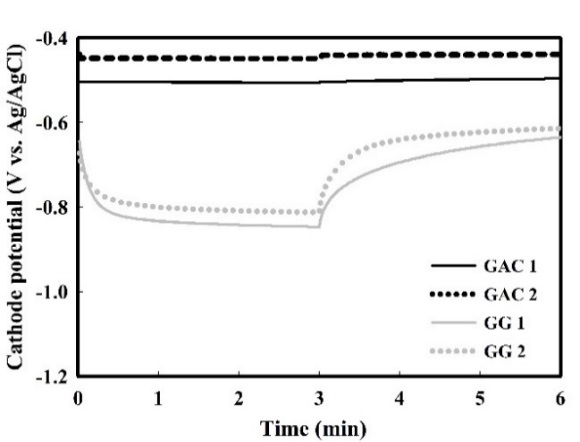
(e) (f)

Figure S5. Overview of cathode potentials of all biocathodes within one typical cycle (6 min) of intermittent operations at a current density of 10 (a, c and e) and 35 A/m^2^cat_proj_ (b, d, and f). The cathode potentials of GG biocathodes fluctuated with -0.8 V to -1.0 V during the period with current time-ON, and -0.6 V to -0.7 V during the period with current time-OFF. However, the cathode potentials of GAC biocathodes kept stable around -0.5 V during each cycle of intermittent operation.

**F. Switching from galvanostatic control to potentiostatic control of all biocathodes**

During the stable performance period at a current density of 10 A/m^2^ cat_proj_, the control strategy of all biocathodes was switched from galvanostatic control to potentiostatic control for about 110 hours, with fixed cathode potentials of -0.5 V for GAC biocathodes and -0.9 V for GG biocathodes. The current densities generated by all these biocathodes were around 10 A/m^2^ cat_proj_. In addition, the current-to-methane efficiencies for all the biocathodes were also quite similar (61% for GAC_1_, 53% for GAC_2_, 52% for GG_1_ and 52% for GG_2_) as that obtained in the galvanostatic control with 10 A/m^2^ cat_proj_. This shows that biocathodes have similar performance under galvanostatic and potentiostatic control.

Figure S6. Overview of biocathodes performance under potentiostatic control with fixed cathode potentials of -0.5 V for GAC biocathodes and -0.9 V for GG biocathodes.

**References**

Parada, A.E., Needham, D.M. and Fuhrman, J.A. (2015) Every base matters: assessing small subunit rRNA primers for marine microbiomes with mock communities, time series and global field samples. Environmental Microbiology.

Wang, Y. and Qian, P.-Y. (2009) Conservative fragments in bacterial 16S rRNA genes and primer design for 16S ribosomal DNA amplicons in metagenomic studies. PLoS One 4(10), e7401.

Kvist, T., Ahring, B.K. and Westermann, P. (2007) Archaeal diversity in Icelandic hot springs. Fems Microbiology Ecology 59(1), 71-80.

Hamady, M., Walker, J.J., Harris, J.K., Gold, N.J. and Knight, R. (2008) Error-correcting barcoded primers allow hundreds of samples to be pyrosequenced in multiplex. Nature methods 5(3), 235.

Ramiro-Garcia, J., Hermes, G.D., Giatsis, C., Sipkema, D., Zoetendal, E.G., Schaap, P.J. and Smidt, H. (2016) NG-Tax, a highly accurate and validated pipeline for analysis of 16S rRNA amplicons from complex biomes. F1000Research 5.

Quast, C., Pruesse, E., Yilmaz, P., Gerken, J., Schweer, T., Yarza, P., Peplies, J. and Glöckner, F.O. (2012) The SILVA ribosomal RNA gene database project: improved data processing and web-based tools. Nucleic Acids Research, gks1219.

Somerfield, P.J. (2008) Identification of the Bray-Curtis similarity index: Comment on Yoshioka (2008). Marine Ecology Progress Series 372, 303-306.
